# Validation of an Arabic Instrument for the Assessment of Patient-Centered Care Among Patients with Diabetes in the Primary Healthcare Setting in the Kingdom of Saudi Arabia

**DOI:** 10.3390/healthcare14162510

**Published:** 2026-08-12

**Authors:** Nizar Alsubahi, Milena Pavlova, Mohannad Alkhateeb, Sami Alanazi, Wim Groot

**Affiliations:** 1 Department of Health Service and Hospital Administration, Faculty of Economics and Administration, King Abdul Aziz University, Jeddah 21589, Saudi Arabia; 2 Department of Health Services Research, CAPHRI, Maastricht University Medical Center, Faculty of Health, Medicine and Life Sciences, 6200 MD Maastricht, The Netherlands; 3 School of Public Health and Community Medicine, Institute of Medicine, University of Gothenburg, 40530 Gothenburg, Sweden; 4 Department of Managing Health Services and Hospitals, Faculty of Business, King Abdulaziz University, Rabigh 21911, Saudi Arabia; 5 Maastricht Graduate School of Governance, Maastricht University, Boschstraat 24, 6211 AX Maastricht, The Netherlands; 6 Maastricht Economic and Social Research Institute on Innovation and Technology, United Nations University, 6200 MD Maastricht, The Netherlands

**Keywords:** diabetics, primary care, patient-centered care, psychometrics, Arabic healthcare instrument, questionnaire translation

## Abstract

**Background:** Patient-centered care is a fundamental element of effective diabetes management; however, validated Arabic instruments assessing this concept are currently lacking in Arab countries. This study examined the validity and reliability of the Arabic version of the patient-centered care instrument among patients with diabetes. **Methods:** A face-to-face administered questionnaire among 594 patients with diabetes in Saudi Arabia between July and August 2023. The questionnaire consisted of 36 items that assessed patient-centered care (PCC-36). PCC-36 was translated into Arabic in accordance with the World Health Organization guidelines for the translation of instruments before psychometric evaluation. For each PCC-36 function, confirmatory factor analysis was conducted, and Cronbach’s alpha coefficients were used to test the reliability and internal consistency of the measures. The data were analyzed using SPSS version 28 and AMOS version 28. **Results:** The Arabic translation of PCC-36 showed high interitem correlations, and confirmatory factor analysis indicated an acceptable fit to the data (CFI = 0.936, GFI = 0.90, CMIN/DF = 2.42, RMSEA = 0.079). Cronbach’s alpha confirmed the internal consistency of the eight PCC-36 functions, with an alpha value of 0.96. **Conclusions:** This study demonstrates that the Arabic version of PCC-36 is a valid and reliable instrument for measuring patient-centered care among patients with diabetes in Saudi Arabia. However, further validation in other Arab countries and healthcare settings is recommended.

## 1. Introduction

Patient-centered care (PCC) has emerged as an important approach in diabetes management. Patients with diabetes are more likely to experience adverse health outcomes [1,2], such as uncontrolled blood glucose levels, diabetes-related complications, and higher mortality rates overall [3]. PCC plays a vital role in addressing patients’ cognitive and affective needs, going beyond the physical aspects of diabetes management. In recent decades, the occurrence of diabetes has gradually and continuously grown, leading to an estimated 422 million individuals worldwide currently living with the condition [4]. Furthermore, projections indicate that the number of people with diabetes will double by 2030 [5,6].

The insufficiency of health services, coupled with the rising prevalence of diabetes, contributes to an unequivocally suboptimal provision of diabetes care, despite the presence of numerous medical centers, consultant clinics, and associations dedicated to addressing this chronic condition. Recent national statistics from several Arab countries highlight the frequency of diabetes in these populations: 13.9% in Iraq [7,8]; 5.4% among individuals aged 20 to 79 in Yemen [9]; 15.3% in Gaza, the West Bank, and East Jerusalem in Palestine [10]; 12.6% in Syria [11]; and 14.6% in Lebanon [12].

Previous research has indicated that adopting a PCC approach requires substantial transformations across high-, middle-, and low-income countries to enhance the quality of healthcare services. High-income European countries have adopted diverse approaches and tools to facilitate PCC implementation. In the Middle Eastern context, PCC implementation is typically preceded by efforts to assess the extent to which healthcare settings promote a person-centered climate. These evaluations serve as a foundation for tailoring PCC strategies that address context-specific barriers and cultural factors. However, despite its significance, PCC has received limited attention from the scientific community and regulatory bodies, and is subject to controversies among healthcare providers, professionals, and decision-makers on a global scale [13].

**Figure 1:**
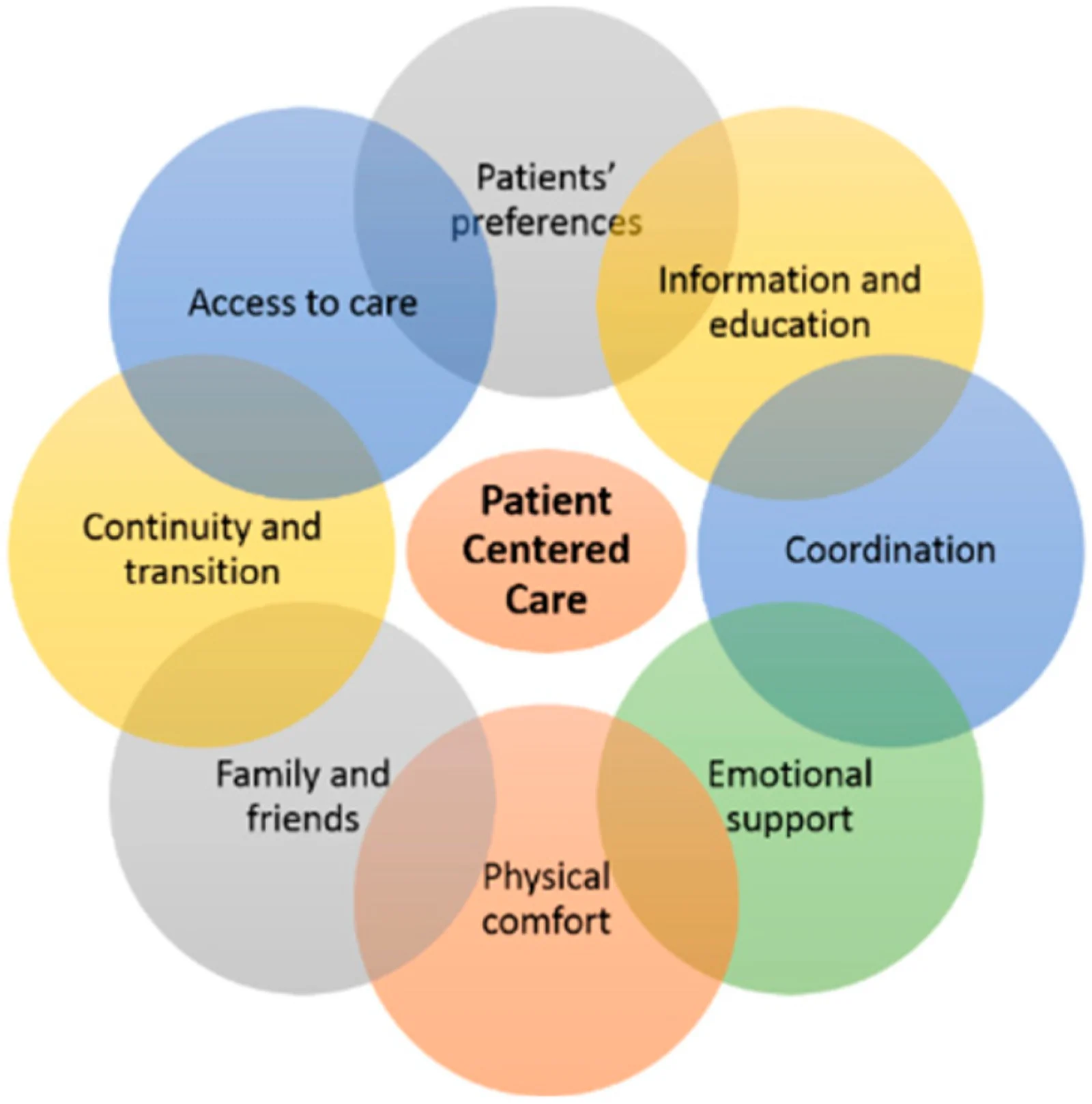
Framework of the eight dimensions of patient-centered care, as defined by the Picker Institute [13].

In collaboration with the Picker Institute, the Harvard School of Medicine conducted an extensive study to investigate the components of PCC [14,15]. They identified eight dimensions of PCC (Figure 1): respecting patients’ values, preferences, and expressed needs; providing information and education; ensuring access to care; offering emotional support to alleviate fear and anxiety; involving family and friends; ensuring continuity and smooth transitions between healthcare settings; addressing physical comfort; and coordinating care effectively [14,16]. The 36-item PCC questionnaire, commonly referred to as PCC-36, covers these eight dimensions.

Despite its significance, the translation of the PCC-36 questionnaire into languages other than English and the evaluation of its psychometric properties have been limited. The English version of PCC-36 has been established as a valid and reliable instrument to assess PCC in the context of multimorbidity [16] and diabetes [17]. Several studies have validated Arabic tools that evaluate patient-centered care and communication in healthcare settings, such as the Person-Centered Climate Questionnaire–Patient Version [18] and the Patient-Centered Communication instrument [19]. However, there is limited validation of these tools specifically in patients with diabetes, despite the crucial role of patient-centered strategies in managing chronic diseases. Prior studies have not validated the PCC-36 instrument specifically in patients with diabetes. Filling this gap is essential because the management of diabetes depends on ongoing involvement, shared choices, and clear communication—fundamental aspects of PCC. Nevertheless, validation of such instruments among patients with diabetes remains limited, despite the importance of patient-centered approaches in managing chronic diseases. No study to date has translated and validated PCC-36 in Arabic specifically for use among populations with diabetes in Arab countries. Therefore, this study aimed to assess the validity and reliability of the Arabic version of PCC-36, a measure of PCC in patients with diabetes.

## 2. Method

### 2.1. Study Design and Setting

This observational study was conducted between 1 July 2023 and 29 August 2023 at five prominent referral healthcare facilities in Jeddah, Saudi Arabia. These included the primary healthcare centers associated with King Fahad Hospital and King Abdullah Medical City, as well as Al-Thaghr Hospital, East Jeddah Hospital, and King Abdulaziz Hospital. All primary health centers provide services for patients with diabetes.

### 2.2. Participants and Procedure

The study took place at 47 primary healthcare centers (PHCs). Within each center, simple random sampling was used. Every day, a random list of eligible patients was created from the appointment records, and about 10–15 patients with diabetes were invited for face-to-face interviews. Eligibility was assessed in advance using the established inclusion and exclusion criteria. Participants were eligible if they were at least 18 years old, as adults can give informed consent and accurately describe their healthcare experiences. The study involved individuals diagnosed with either type 1, type 2, or gestational diabetes who were clinically stable and visiting PHCs during data collection. Patients with diabetes complications such as neuropathy, retinopathy, nephropathy, or high HbA1c levels were eligible if their conditions were stable enough for outpatient care. Exclusion criteria included cognitive impairment, communication issues, or language barriers that could hinder interview completion, ensuring the data’s quality and reliability. Data collection was carried out by the research team, comprising the study authors and trained research assistants. All data collectors were qualified healthcare professionals with prior experience in administering structured questionnaires and conducting face-to-face interviews. The interview protocol was based on the instrument developed in our previous study [17]. Before commencing data collection, all team members received standardized training on the study procedures and interview protocol to ensure consistency across data collection sites. Participants were interviewed individually in private rooms at participating PHCs. Convenience sampling was employed to recruit participants and enhance the representativeness of the study sample. Responses were recorded electronically via the Qualtrics platform. During data collection, the team held regular meetings to monitor progress, address operational challenges, and ensure compliance with the study protocol. These meetings helped maintain data consistency and quality. Interviews were conducted in private rooms to safeguard participants’ confidentiality and foster honest responses. Furthermore, standardized training for interviewers helped minimize bias and ensured consistent data collection methods across all primary healthcare centers involved.

### 2.3. Study Tool

The survey encompassed a range of questions, including both sociodemographic data and the patient-centered care instrument. The patient-centered care 36-item instrument was originally developed and psychometrically validated in an earlier study [17]. It includes 36 items grouped into eight essential dimensions of patient-centered care. These dimensions evaluate patient preferences (7 items), physical comfort (5 items), care coordination (4 items), continuity and transitions (4 items), emotional support (4 items), access to care (5 items), information and education (4 items), and family and friends’ involvement (3 items). A 5-point Likert scale is used to obtain patients’ responses to 36 items assessing their perceptions of the quality of care (very good, good, neutral, poor, and very poor). Higher scores reflect more positive perceptions among patients.

### 2.4. Translating the PCC-36 Instrument

Before translating the PCC-36 instrument into Arabic, we obtained permission from the original instrument developer. The PCC-36 instrument was translated in accordance with the World Health Organization (WHO) guidelines for instrument translation. At first, the main researcher translated the instrument into Arabic, using simple language to facilitate patient comprehension. Afterward, this Arabic version of PCC-36 was reviewed by a team of bilingual experts specializing in healthcare and research. Following that, a different translator, unfamiliar with the original tool, retranslated it into English. As a result of this step, any discrepancies or misunderstandings in the translation were identified and rectified.

**Figure 2:**
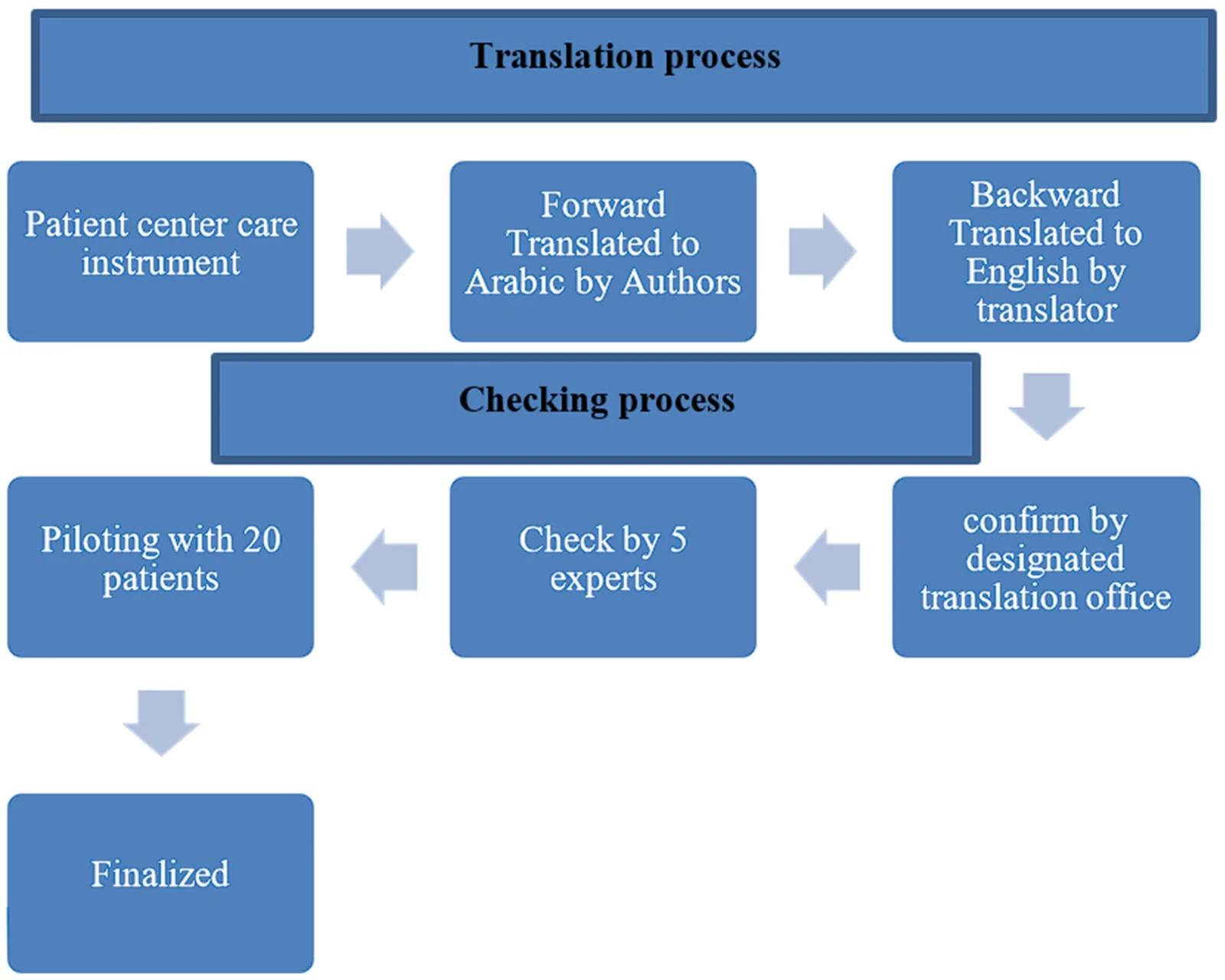
Translation process in accordance with World Health Organization guidelines.

A pre-test study was conducted with 20 participants who fulfilled the study’s inclusion criteria (see Figure 2). To confirm that the respondents accurately reflected the population, participants of different genders, ages, and socioeconomic statuses were included in the pre-test study. These participants provided feedback on the wording, meaning, format, and length of the survey items during the pilot phase. To verify consistency, their written responses to the PCC-36 items were compared with their verbal responses [20]. Following their feedback, the instrument was modified to improve clarity and accuracy. The translated instrument can be found in the Supplementary File.

### 2.5. Data Analysis

Descriptive statistics include means and standard deviations used to summarize continuous variables, as well as frequencies and percentages for categorical variables. Confirmatory factor analysis (CFA) was conducted to evaluate the construct validity of PCC-36. Bryant and Yarnold [21] described CFA as a widely accepted method for testing the degree to which measured variables are representative of their intended constructs, thereby confirming or refuting the underlying measurement theory. The CFA results revealed an acceptable fit of the data and demonstrated that PCC-36 measures the same construct as the expected theoretical model. The CFA results support the validity of PCC-36. Model fit was assessed using the Comparative Fit Index (CFI), Goodness-of-Fit Index (GFI), Chi-square/degrees of freedom ratio (CMIN/DF), and Root Mean Square Error of Approximation (RMSEA). Standardized factor loadings were analyzed to assess the relationship between each observed item and its corresponding latent construct. SPSS version 28 and AMOS version 28 were used to analyze the data.

### 2.6. Ethical Consideration

Ethical approval was obtained from the Central Institutional Review Board (IRB) at the Saudi Arabia Ministry of Health (log number: A01569) and the Ethics Review Committee at the Faculty of Health, Medicine & Life Sciences at Maastricht University in accordance with the relevant guidelines and regulations in Ethics Approval and Consent to participate as described in the Declaration of Helsinki (approval number: FHML-REC/2023/045). All participants provided electronic informed consent before their involvement. During this process, patients’ national IDs were verified to ensure eligibility and prevent duplicate entries. Identifying information was solely used for eligibility checks and not saved in the dataset. Responses were collected anonymously, and all data were kept confidential and securely stored on password-protected servers accessible only to the principal researcher.

## 3. Results

### 3.1. Participants

A total of 809 eligible patients were invited to participate in the study. Of these, 594 completed the questionnaire, yielding a response rate of 73.4%. The other 215 eligible patients opted out, mainly due to time constraints or scheduling conflicts. Only fully completed questionnaires were used in the psychometric analyses, and any with a significant amount of missing data were excluded. Among the 594 participants, the distribution by sex was nearly equal, with 49.3% males and 50.7% females. The largest age group comprised individuals aged 41–60 years (42.8%, n = 254). Regarding educational attainment, 43.3% had completed secondary school or a lower level of education. Most participants were married (68.0%), were unemployed (45.3%), and reported a monthly income of 5000 Saudi Riyals or less (60.9%). In addition, most were Saudi nationals (85.4%), nearly three-quarters were non-smokers (72.6%), and 44.4% had been living with diabetes for more than 15 years. The results also indicate that 68.4% of the participants had type 2 diabetes, 44.4% were overweight, and 93.4% were in good health (Table 1).

### 3.2. Construct Validity

**Figure 3:**
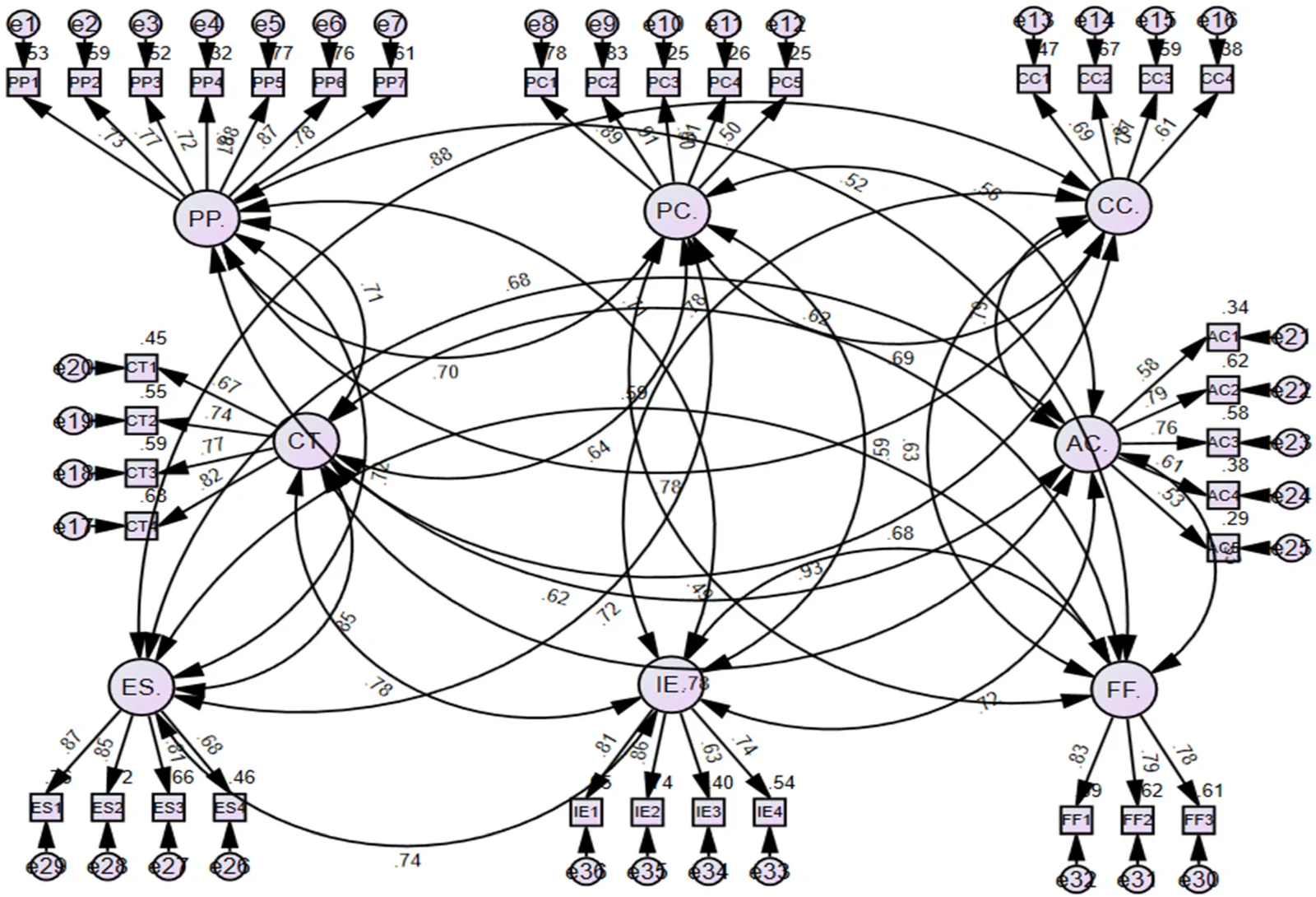
Confirmatory factor analysis of 36 items of patient-centered care.

Figure 3 shows that confirmatory factor analysis (CFA) was conducted to evaluate the construct validity of the Arabic PCC-36. The overall model demonstrated an acceptable fit to the data (χ^2^ = 2729.412, df = 566, *p* < 0.001; χ^2^/df = 4.822; CFI = 0.849; TLI = 0.832; RMSEA = 0.080, 90% CI = 0.077–0.083; PCLOSE < 0.001; SRMR = 0.077). Although the CFI and TLI values were slightly below the conventional threshold of 0.90, the RMSEA and SRMR values indicated an acceptable model fit, supporting the proposed eight-factor structure of the Arabic PCC-36. Standardized factor loadings are presented in Table 2, regression weights in Table 3, and convergent validity indices (CR and AVE) in Table 4.

Construct validity received additional support from the factor loading estimates, as shown in Table 3, and all freely estimated loadings were statistically significant (*p* < 0.001). Most items showed moderate-to-high loadings on their respective latent constructs. However, some items, especially in the access to care domain, exhibited comparatively lower loadings. Despite this, they were retained because they are part of the original PCC-36 measurement model and help maintain the overall measurement structure.

To further evaluate the measurement properties of the Arabic PCC-36, composite reliability (CR) and average variance extracted (AVE) were calculated for each construct. As shown in Table 4, all constructs demonstrated CR values greater than the recommended threshold of 0.70 and AVE values of 0.50 or higher, indicating satisfactory internal consistency and providing additional evidence of convergent validity.

### 3.3. Reliability Analysis

Cronbach’s alpha was used to evaluate the tool’s reliability and internal consistency [22]. As shown in Table 5, there was high-to-excellent reliability across all eight domains and the overall scale. Cronbach’s alpha was 0.96 for the overall PCC-36 instrument. In particular, patient preferences, physical comfort, coordination of care, continuation and transition, emotional support, access to care, information and education, and family and friends exhibited alpha coefficients of 0.89, 0.82, 0.79, 0.82, 0.87, 0.77, 0.82, and 0.83, demonstrating strong internal consistency across all domains. Table 5 is presented below.

## 4. Discussion

### 4.1. Main Findings

The PCC-36 instrument measures PCC for patients with multimorbidity [16] and diabetes [17], and it has been widely used [23,24]. Nevertheless, validity testing was limited to English. This study validated the Arabic version of the PCC-36 instrument among patients with diabetes, a group requiring continuous, coordinated, and person-centered care. The PCC-36 instrument was translated based on guidelines provided by the World Health Organization for culturally adapting and translating original tools [20]. According to the guidelines, both forward and backward translations were conducted to ensure semantic equivalence. A culturally equivalent instrument is achieved by using this approach when translating an existing tool [25].

The present study included 594 patients with diabetes who received care at primary healthcare centers in Saudi Arabia. The construct validity of the Arabic PCC-36 was evaluated using CFA, a widely accepted approach for examining whether the observed data support the hypothesized measurement model. As described by Cronbach and Meehl [26], construct validity reflects the extent to which an instrument accurately measures the theoretical construct that it is intended to assess. The CFA findings demonstrated that the proposed eight-domain structure of the Arabic PCC-36 had an acceptable fit to the data, with all items loading significantly on their respective latent constructs, thereby supporting the instrument’s construct validity.

The Arabic version of PCC-36 showed excellent internal consistency, with a Cronbach’s alpha of 0.96. This suggests that the tool reliably assesses patient-centered care across its eight domains. Although alpha values above 0.90 are usually considered indicative of excellent reliability, they may also suggest redundancy among items targeting similar aspects of the construct. In this study, the high alpha value is likely due to the comprehensive design of the original 36-item instrument, which was used without changes to its conceptual framework. Keeping the original structure allows for comparison with previous validation studies and preserves the instrument’s content validity.

The internal consistency of PCC-36 has been evaluated in different healthcare contexts. Previous studies have validated the instrument among patients with multimorbidity in the Netherlands [23] and, more recently, among patients with diabetes in Saudi Arabia [17]. The original English version achieved an overall Cronbach’s alpha coefficient of 0.89, whereas the Arabic version achieved an overall alpha of 0.96. These findings indicate excellent internal consistency of the Arabic PCC-36, suggesting that its items consistently measure the underlying construct of patient-centered care. Although Cronbach’s alpha values above 0.90 may indicate some overlap among items, the high reliability observed in this study supports the stability of the instrument while preserving the original conceptual framework of PCC-36 [26]. Most items showed satisfactory factor loadings, but a few, especially in the access to care domain, had lower loadings. These items were kept because they are vital parts of the original PCC-36 framework and support the instrument’s content validity. The lower loadings might be due to differences in how primary healthcare is organized and delivered, patients’ expectations regarding access, or cultural factors that shape how these items are understood in the Saudi healthcare context. Keeping these items ensures consistency with the original tool and allows for better comparison across validation studies. Future research should examine how these items perform in other Arabic-speaking populations and healthcare environments. Beyond the CFA results, the CR and AVE values reinforced the reliability and validity of the Arabic PCC-36. All constructs had CR values exceeding 0.70 and AVE values of at least 0.50, demonstrating good convergent validity and indicating that the items within each domain effectively reflect their respective latent constructs. These results further confirm the strong psychometric qualities of the Arabic version of PCC-36.

### 4.2. Strengths and Limitations

To the best of our knowledge, this is the first study of its kind to be conducted in the broader Arab region. There is a limitation to consider: most participants were Saudi Arabian; therefore, further testing of the Arabic-translated tool is necessary in other Arab countries to ensure its generalizability. We also recommend conducting further research to evaluate the tool’s effectiveness in different contexts. It is important to assess the tool’s effectiveness in different contexts to ensure that it applies to other cultures. Finally, further research into the tool’s effectiveness is needed to determine its long-term impact.

### 4.3. Implications for Policy, Practice, and Research

Patients with diabetes require continuous and comprehensive medical care, as well as a variety of management strategies [27]. PCC-36 is an instrument designed to assess PCC in primary care among patients with chronic disease [16,17]. It has been reported that there is limited research on the PCC needs of patients with diabetes in Arab countries, including Saudi Arabia [28]. More research is needed. Furthermore, it is expected that the prevalence of diabetic care in Saudi Arabia will double in the next two decades [29]. The healthcare system in Saudi Arabia is undergoing continuous transformation to become more patient-centered and value-based [30,31]. This transformation represents an important step on the road to improving the quality of healthcare services. It can contribute to achieving the 2030 Saudi Vision, which aims to improve the quality of care across all healthcare facilities in Saudi Arabia [32]. When examining patient-centered care in individuals with diabetes, it is important to consider health literacy. Effective communication, patient education, and patients’ ability to understand and use health information are key to successful diabetes management. Those with limited health literacy often struggle to understand treatment plans, engage in shared decision-making, and navigate healthcare systems, which can affect their perceptions of patient-centered care. Recent research shows that poor health literacy, especially when combined with complex care needs, is associated with worse clinical outcomes, including higher mortality, more hospital readmissions, and increased emergency visits [33]. These results highlight the need for valid, culturally sensitive tools to assess patient-centered care, helping identify where communication and patient engagement can be improved to enhance health outcomes.

The Arabic version showed strong psychometric qualities among patients with diabetes at primary healthcare centers in Saudi Arabia. While the instrument could be used in other Arabic-speaking populations, additional validation across various Arab countries and healthcare settings is necessary to confirm its broader applicability.

## 5. Conclusions

PCC-36, developed by the Harvard School of Medicine and the Picker Institute, is intended to identify eight fundamental dimensions of PCC. This study involved translating the instrument into Arabic in accordance with WHO guidelines, followed by a psychometric evaluation. To the best of our knowledge, this is the first study to evaluate the validity and reliability of the PCC-36 instrument in an Arab context. The Arabic version of the PCC-36 instrument demonstrated robust psychometric properties, and the scale provided reliable and valid measurements of diabetic patients in Saudi Arabia. Nonetheless, further studies are needed to investigate the psychometric properties of the PCC-36 instrument in Arabic and in other contexts.

## Figures and Tables

**Table 1:** Sociodemographic characteristics of the respondents (N = 594).

Demographic Characteristics	Category	Frequency (n)	Percent (%)
Age (years)	20 or less	35	5.9
	21–40	121	20.4
	41–60	254	42.8
	Over 60	184	31.0
Gender	Male	293	49.3
	Female	301	50.7
Educational level	Illiterate	99	16.7
	Secondary school or less	257	43.3
	Diploma	43	7.2
	Bachelor	157	26.4
	Postgraduate	38	6.4
Marital status	Married	404	68.0
	Single	101	17.0
	Divorced	35	5.9
	Widower	54	9.1
Employment status	Unemployed	269	45.3
	Employed	152	25.6
	Self-employed	33	5.6
	Retired	140	23.6
Monthly income	5000 SR or less	362	60.9
	5001–10,000 SR	110	18.5
	10,001–15,000 SR	62	10.4
	More than 15,000 SR	60	10.1
Nationality	Saudi	507	85.4
	Non-Saudi	87	14.6
Smoking	Smoker	79	13.3
	Previous smoker	84	14.1
	Non-smoker	431	72.6
Diagnosis of diabetes	Less than 6 years	30	5.1
	6–10 years	209	35.2
	11–15 years	91	15.3
	Over 15 years	264	44.4
Type of diabetes	Type 1	169	28.5
	Type 2	406	68.4
	Gestational	19	3.2
Body mass index (BMI)	Underweight	17	2.9
	Healthy weight	138	23.2
	Overweight	175	29.5
	Obese	264	44.4
Health state	Stable	555	93.4
	Severe	39	6.6

**Table 2:** Confirmatory factor analysis, mean (standard deviation), and item–total correlation.

Patient-Centered Care Items 5-Point Likert Scale: 1 = Very Poor, 2 = Poor, 3 = Neutral, 4 = Good, 5 = Very Good	Mean (SD)	Item–Total Correlation	B
**1. PP**			
1.1 PP1	1.66 (0.889)	0.533	0.73
1.2 PP2	1.92 (1.033)	0.592	0.77
1.3. PP3	1.96 (1.066)	0.520	0.72
1.4. PP4	2.30 (1.317)	0.321	0.88
1.5. PP5	1.85 (1.008)	0.775	0.87
1.6. PP6	1.83 (1.006)	0.765	0.87
1.7. PP7	1.74 (0.997)	0.601	0.84
**2. PC**			
2.1 PC1	1.95 (1.049)	0.473	0.89
2.2 PC2	2.00 (1.110)	0.518	0.81
2.3 PC3	1.60 (0.799)	0.533	0.81
2.4 PC4	1.80 (1.019)	0.515	0.50
2.5 PC5	1.61 (0.849)	0.438	0.82
**3. CC**			
3.1 CC1	1.87 (1.043)	0.498	0.69
3.2 CC2	1.86 (0.971)	0.688	0.62
3.3 CC3	2.08 (1.123)	0.563	0.61
3.4 CC4	2.52(1.303)	0.354	0.62
**4. CT**			
4.1 CT1	2.14 (1.107)	0.422	0.82
4.2 CT2	1.75 (0.900)	0.567	0.77
4.3 CT3	1.89 (0.956)	0.588	0.74
4.5 CT4	1.88 (0.936)	0.676	0.76
**5. ES**			
5.1 ES1	2.12 (1.091)	0.760	0.87
5.2 ES2	2.05 (1.083)	0.725	0.85
5.3 ES3	2.34 (1.195)	0.654	0.81
5.4 ES4	2.08 (1.195)	0.456	0.68
**6. AC**			
6.1 AC1	1.87 (1.109)	0.388	0.34
6.2 AC2	1.79 (0.952)	0.635	0.62
6.3 AC3	2.04 (1.210)	0.578	0.58
6.4 AC4	2.47 (1.310)	0.367	0.38
6.5 AC5	1.96(1.076)	0.279	0.29
**7. IE**			
7.1 IE1	1.83 (1.006)	0.651	0.55
7.2 IE2	1.74 (0.997)	0.731	0.54
7.3 IE3	1.95 (1.049)	0.396	0.40
7.4 IE4	2.00 (1.110)	0.553	0.54
**8. FF**			
8.1 FF1	1.84(1.015)	0.685	0.69
8.2 FF2	1.68 (0.894)	0.608	0.62
8.3 FF3	1.81 (0.987)	0.625	0.61

PP: patient preference; PC: physical comfort; CC: coordination of care; CT: continuity and transition; ES: emotional support; AC: access to care; IE: information and education; FF: family and friends.

**Table 3:** Confirmatory factor analysis results: regression weights (factor loadings) for the Arabic PCC-36 measurement model.

Path	Std. Error	*p*
PP <- PCC	0.048	***
PC <- PCC	0.053	***
CC <- PCC	0.055	***
AC <- PCC	0.058	***
FF <- PCC	0.049	***
IE <- PCC	0.046	***
ES <- PCC		
CT <- PCC	0.046	***
PP1 <- PP	0.034	***
PP2 <- PP	0.039	***
PP3 <- PP	0.041	***
PP4 <- PP	0.056	***
PP5 <- PP		
PP6 <- PP	0.034	***
PP7 <- PP	0.037	***
PC1 <- PC	0.058	***
PC2 <- PC		
PC3 <- PC	0.045	***
PC4 <- PC	0.057	***
PC5 <- PC	0.047	***
CC1 <- CC	0.051	***
CC2 <- CC	0.047	***
CC3 <- CC		
CC4 <- CC	0.065	***
CT4 <- CT		
CT3 <- CT	0.046	***
CT2 <- CT	0.044	***
CT1 <- CT	0.056	***
AC1 <- AC	0.053	***
AC2 <- AC	0.045	***
AC3 <- AC		
AC4 <- AC	0.062	***
AC5 <- AC	0.051	***
ES4 <- ES	0.048	***
ES3 <- ES		
ES2 <- ES	0.041	***
ES1 <- ES	0.040	***
FF3 <- FF	0.047	***
FF2 <- FF	0.043	***
FF1 <- FF		
IE4 <- IE	0.037	***
IE3 <- IE	0.057	***
IE2 <- IE		
IE1 <- IE	0.038	***

*** *p*-value less than 0.001.

**Table 4:** Composite reliability and convergent validity.

Path			Estimate	(AVE)	(CR)
PP1	<---	PP.	0.731	0.59	0.90
PP2	<---	PP.	0.767		
PP3	<---	PP.	0.720		
PP4	<---	PP.	0.569		
PP5	<---	PP.	0.880		
PP6	<---	PP.	0.873		
PP7	<---	PP.	0.778		
PC1	<---	PC.	0.885	0.50	0.83
PC2	<---	PC.	0.911		
PC3	<---	PC.	0.500		
PC4	<---	PC.	0.511		
PC5	<---	PC.	0.500		
CC1	<---	CC.	0.686	0.53	0.80
CC2	<---	CC.	0.816		
CC3	<---	CC.	0.768		
CC4	<---	CC.	0.615		
CT4	<---	CT.	0.822	0.56	0.83
CT3	<---	CT.	0.768		
CT2	<---	CT.	0.740		
CT1	<---	CT.	0.668		
AC1	<---	AC.	0.579	0.50	0.87
AC2	<---	AC.	0.788		
AC3	<---	AC.	0.762		
AC4	<---	AC.	0.613		
AC5	<---	AC.	0.534		
ES4	<---	ES.	0.682	0.65	0.80
ES3	<---	ES.	0.810		
ES2	<---	ES.	0.846		
ES1	<---	ES.	0.872		
FF3	<---	FF.	0.780	0.64	0.82
FF2	<---	FF.	0.790		
FF1	<---	FF.	0.829		
IE4	<---	IE.	0.737	0.58	0.84
IE3	<---	IE.	0.631		
IE2	<---	IE.	0.858		
IE1	<---	IE.	0.809		

**Table 5:** Reliability of patient-centered care variables according to Cronbach’s alpha.

Variable	Number of Items	Original Version’s Cronbach’s Alpha	Translated Version’s Cronbach’s Alpha
Patient preferences	7	0.92	0.89
Physical comfort	5	0.72	0.82
Coordination of care	4	0.82	0.79
Continuity and transition	4	0.87	0.82
Emotional support	4	0.74	0.87
Access to care	5	0.80	0.77
Information and education	4	0.78	0.82
Family and friends	3	0.92	0.83
Total patient-centered care variables	36	0.89	0.96

## Data Availability

The data presented in this study are available on request from the corresponding author. The data are not publicly available due to privacy or ethical restrictions.

## Supplementary Materials

The following supporting information can be downloaded at: https://www.mdpi.com/article/10.3390/healthcare14162510/s1, Supplementary File: The Arabic version of the tool assessing patient-centered care: the 36-item patient-centered primary care instrument.

## Abbreviations

PCC	Patient-Centered Care
PP	Patient Preference
PC	Physical Comfort
CC	Coordination of Care
CT	Continuity and Transition
ES	Emotional Support
AC	Access to Care
IE	Information and Education
FF	Family and Friends
