# Supplementary material for: Validation of an Arabic Instrument for the Assessment of Patient-Centered Care Among Patients with Diabetes in the Primary Healthcare Setting in the Kingdom of Saudi Arabia
Source: Healthcare (Basel). 2026 Aug 12;14(16):2510. doi: 10.3390/healthcare14162510 (PMC13512653; doi:10.3390/healthcare14162510)
Supplement: Supplementary file 1 [file healthcare-14-02510-s001.zip › healthcare-4451867-supplementary.pdf]

The Arabic version of the tool

Patient-centered care: the 36-item patient-centered primary care instrument

Patient-Centered Care PCC	Very good 5	Good 4	Neutral 3	Poor 2	Very poor 1
**Patients' preferences**					
1. I felt taken seriously					
2.My wishes and preferences were taken into account when choosing a treatment					
3.I was involved in decisions about my treatment					
4.The influence that the treatment can have on my life was taken into account					
5. I was helped to determine my own treatment goals					
6.I felt supported to achieve my treatment goals					
7. I received advice that I really could use					
**Physical comfort**					
8. Attention was given to my physical comfort (such as the management of pain, shortness of breath)					
9. Attention was paid to fatigue and insomnia					
10. The (waiting) rooms were clean					
11. The (waiting) rooms were comfortable					
12. In the treatment room(s) and at the counter there was sufficient privacy					
**Coordination of care**					
13. Everyone was well informed; I only had to tell my story once					
14. The care was well attuned between the practitioners involved					
15. I knew who was coordinating my care					
16. I could easily contact someone with questions					
**Continuity and transition**					
17. When being referred to another care provider (specialist /dietician/ physiotherapist) I was well informed about where to go and why					
18. With a referral, all my information was passed on correctly					
19. Advice (such as medication) from different practitioners (medical specialists and family doctor) was well attuned to each other					
20. The treatment of the family doctor is in line with the treatment of other care providers					
**Emotional support**					
21. Emotional support was also provided					
22. Attention was paid to possible feelings of fear, gloom and anxiety					
23. I was made aware of the possibilities for more intensive emotional support					
24. Attention was paid to the impact of my health on my private life (family, relatives, work, social life)					
**Access to care**					
25. It was no problem to go from my home to my family doctor and back again					
26. The general practice was easily accessible					
27. I could easily schedule an appointment quickly					
28. On a visit I didn't have to wait long before it was my turn					
29. I could easily request a repeat recipe					
**Information and education**					
30. I was well informed					
31. The information I received was well explained					
32. I had easy access to my own data (lab results, medication overview, referrals)					
33. I could ask all the questions I wanted					
**Family and friends**					
34. With my consent, relatives were involved in my treatment					
35. Attention was given to care and support provided by family members					
36. Attention was given to possible questions from my family members					

الرعاية التي تركز على المريض

العبارة	أوافق بشدة 5	أوافق 4	محايد 3	أرفض 2	أرفض بشدة 1
**أ- فيما يخص تفضيلاتك**					
1. شعرت بالإهتمام في العيادة					
2. تمت إستشارتي في تقييم خطتي العلاجية					
3. شاركت في اتخاذ القرارات المتعلقة بخطة علاجي					
4. تم إخباري بالآثار الجانبية للعلاج					
5. تمت مساعدتي في تحديد أهداف علاجي					
6. شعرت بالدعم لتحقيق أهدافي العلاجية					
7. تلقيت نصائح يمكنني أن أستخدمها فعلا					
**ب- فيما يخص راحتك البدنية**					
8. تم الإهتمام لراحتي الجسدية (علاج الألم وضيق التنفس)					
9. تم الإهتمام لراحتي النفسية (التعب، قلة النوم)					
10. كانت غرف (الإنتظار) نظيفة					
11. كانت غرف (الإنتظار) مريحة					
12. كانت هناك خصوصية كافية في العيادة					
**ج – تنسيق الرعاية**					
13. كان الجميع على دراية جيدة بحالتي الصحية؛ كان علي فقط أن أحكي سيرتي المرضية مرة واحدة					
14. كان هناك تنسيق جيد بين الممارسين المعنيين					
15. كنت أعرف من المسؤول عن تنسيق رعايتي					
16. يمكنني بسهولة الإتصال بشخص (من المركز الصحي) لطرح الأسئلة (لمتابعة حالتي مثلا)					
**د- فيما يخص الاستمرار والتحويل إلى طبيب اخر**					
17. عند إحالتي إلى مقدم رعاية آخر (أخصائي تغذية / أخصائي علاج طبيعي)، كنت على علم جيد أين أذهب ولماذا					
18. تم تمرير جميع معلوماتي بشكل صحيح وقت الإحالة					
19. كانت المشورة - مثل الدواء - من مختلف الممارسين (الأخصائيين الطبيين وطبيب الأسرة) متوافقة مع بعضها البعض					
20. يتماشى علاج طبيب الأسرة مع علاج مقدمي الرعاية الآخرين					
**هـ – الدعم العاطفي**					
21. تم تقديم الدعم العاطفي بشكل كافي					
22. تم مراعاة مشاعر الخوف والكآبة والقلق لدي					
23. تم إخباري بإمكانيات الدعم العاطفي المكثفة الأخرى					
24. تم إيضاح تأثير صحتي على حياتي الخاصة (الأسرة، الأقارب، العمل، الحياة الإجتماعية)					
**و - فيما يخص إمكانية الوصول إلى الرعاية الصحية**					
25. لم يكن لدي مشكلة في الإنتقال من منزلي إلى طبيب الأسرة والعودة مرة أخرى					
26. من السهل الوصول إلى الطبيب العام					
27. يمكنني بسهولة وبسرعة تحديد موعد					
28. لم يكن علي الإنتظار طويلاً في الزيارة					
29. يمكنني بسهولة طلب وصفة متكررة					
**ن - فيما يخص معلوماتي والتثقيف الصحي لدي**					
30. كنت على علم جيد بحالتي الصحية					
31. تم شرح المعلومات التي تلقيتها بشكل جيد					
32. سهولة الوصول لبياناتي الخاصة (نتائج المختبر، نظرة عامة على الدواء، الإحالات)					
33. يمكنني طرح جميع الأسئلة التي أريدها					
**ي - فيما يخص اهلي وأصدقائي**					
34. شارك (أهلي واصدقائي) في علاجي بموافقتي					
35. قدم (أهلي واصدقائي) الدعم والرعاية الكافية					
36. أجاب الطاقم الطبي على أسئلة عائلتي					
